# The Effects of Education Based on the Nursing Process on Ostomy Self-Care Knowledge and Performance of Elderly Patients with Surgical Stoma

**DOI:** 10.1155/2023/2800796

**Published:** 2023-01-04

**Authors:** Roya Momeni Pour, Azar Darvishpour, Roya Mansour-Ghanaei, Ehsan Kazemnezhad Leyli

**Affiliations:** ^1^Department of Nursing, Zeynab (P.B.U.H) School of Nursing and Midwifery, Guilan University of Medical Sciences, Rasht, Iran; ^2^Social Determinants of Health Research Center, Guilan University of Medical Sciences, Rasht, Iran; ^3^Gastrointestinal and Liver Diseases Research Center, Guilan University of Medical Sciences, Rasht, Iran; ^4^Department of Biostatistics, School of Nursing and Midwifery, Guilan University of Medical Sciences, Rasht, Iran

## Abstract

**Background:**

Patients with surgical stoma experience problems, which can lead to their impaired adaptation and self-efficacy. The nursing process provides a framework for planning and implementing nursing care. This study aimed to investigate the effect of education based on the nursing process on ostomy self-care knowledge and performance of elderly patients with intestinal stoma*. Materials and Methods.* In this quasi experimental study, 52 elderly patients with intestinal ostomy who were referred to Razi Hospital in Rasht and met the inclusion criteria were invited to participate in research. Sampling was done by a simple random method. The intervention group received an educational programme based on the nursing process, whereas the control group received traditional training. The research instruments included a questionnaire to assess the level of ostomy self-care knowledge and ostomy self-care performance. Data were analyzed by SPSS software version 21 using descriptive and inferential statistics at a significant level of *p* < 0.05.

**Results:**

The mean scores of ostomy self-care knowledge and performance in both groups (intervention and control) were increased. However, the improvement in self-care knowledge and performance of the intervention group was significantly greater than that in the control group (*p* < 0.001).

**Conclusions:**

The educational programme based on the nursing process compared to the routine patients training caused more improvement in ostomy self-care knowledge and performance of older adult patients with surgical stoma. Therefore, an educational programme based on the nursing process can be used as an educational model for these patients.

## 1. Background

The world's elderly population is growing rapidly [1]. It is predicted that in the 21st century, the elderly population will reach its highest level in human life, which is why this century has been called the “Century of old age” [2]. Similarly, the report of the general population and housing census of the Islamic Republic of Iran in 2016 in Iran showed that about 9.3% of the country's population consists of people of 60 years and older [3]. In addition, there are some predictions which show that the elderly population of Iran will reach more than 26 million by 2050 [4]. Old age is not a disease. It is the physiological changes that occur over time, and as a result of these changes, the rate of acute and chronic diseases increases [5]. Cancer can be considered an age-related disease because the incidence of most cancers increases with age, rising more rapidly beginning in midlife [6]. Cancer is the second leading cause of death in older adults [7] and among cancers, colorectal cancer has a high prevalence in the elderly. So that approximately 60% of colorectal cancer patients are older than 70, with this incidence likely increasing in the near future [8]. Colorectal cancer in people with colon cancers that have not spread to distant sites is most commonly treated by surgery [9]. Colostomy or ileostomy is a well-known surgical intervention aimed at treatment of various diseases, including cancer [10]. Intestinal ostomy is the creation of an artificial opening in the abdominal wall by surgery that drains the contents of the intestine out of the body [11]. As the frequency of major abdominal operations being performed in older patients rises, there is an associated rise in procedures involving fecal ostomies [12]. Ostomies are being placed frequently in surgically treated elderly patients with colorectal cancer [13]. Studies specifically evaluating the outcome of surgery for colorectal disease in older persons have demonstrated that major procedures should not be denied on the basis of age alone and that colorectal surgery can be conducted with an acceptable margin of safety in older persons [12].

Although the creation of a stoma is associated with positive outcomes such as symptom relief and improvement in overall health, it may have a negative impact on the patient [10]. The majority of patients with stoma suffers with a lack of physical activity, dietary changes, and general lifestyle changes [14]. Ostomy surgery has effects on person's physical appearance and functions and also has a bad effect on patients' body image [15]. Factors linked to living with a stoma may increase or decrease the health-related quality of life after stoma creation. For example, perceptions of living with a stoma are influenced by the presence of stoma complications. Leakage of effluent from the ostomy is perceived as especially bothersome, and a great deal of professional effort goes toward teaching patients to manage their pouching systems in a manner that avoids leakage [10]. To support patients in adjusting to their condition, they should receive self-care-related information and skill training during their hospital stay after colostomy, which might include instructions on colostomy care provided either verbally or via handout [9].

Education has an important part in the development of self-care, independence, and adaptation of individuals to the disease [16]. It has been suggested that patient education might reduce the length of hospital stay, the frequency of postoperative complications, and the frequency of hospital readmissions [17]. Education changes health behaviors and leads to better understanding of the disease and reduces or delays the incidence of complications [18]. Clinical nurses play a major roles to provide the health education for patients in the process of getting used to living with stoma. The main content of the health education should focus on technical skills related to wound care and incontinence (skin cleansing, bag/adapter replacement, changing dressings, and making the proper position). In addition, encouraging people to make decisions about treatment and care, organizing educational programs for daily activities to ensure adaptation to patients' social life, and developing educational materials and educational support systems are recommended [16]. The main goal of nursing care is to provide appropriate quality care to improve the condition of patients [19], and one of the most important policies and principles of nursing care is the use of the nursing process model [20, 21]. This model is a systematic framework for evaluating patients' needs for clinical decision-making. The nursing process moves the care of patients from traditional and old methods to modern, scientific, and patient-centered methods [20]. Patient education is an integral part of the nursing process, and nurses can use this process to assess, plan, implement, and evaluate an effective and individualized patient education programme [22]. However, age-appropriate teaching strategies for the older adult must be planned, purposeful, and adapted to accommodate the special needs of the elderly patient. Specific strategies that adhere to the principles of geragogy should also be an integral part of every nurse's teaching repertoire to promote health literacy in this special population [23].

The effects of education on self-care ability of patients with colorectal cancer have been evaluated in several studies [9, 16, 24]. These studies confirmed the effect of education on patients' self-care ability. It was shown in the study of Wang et al. that multimedia patient education is an adequate educational tool for patients with colorectal cancer who have undergone colostomy surgery [9]. In Shrief and Mokhtar's study, the use of structured educational guidelines had a positive effect on patient knowledge, performance, and self-efficacy regarding colostomy care [24]. In Culha et al.'s study, the effect of self-care education on self-care awareness and agency of patients with colostomy and ileostomy was investigated. These researchers concluded that education may assist in self-care agency and stoma knowledge of patients with stoma [16]. However, the review of the literature indicated that no study has been conducted to investigate the effect of education based on the nursing process on the knowledge and self-care performance of elderly patients with surgical stoma. However, the ultimate goal of patient education programs is to achieve long-lasting changes in behavior by providing patients with knowledge that allows them to make autonomous decisions to take ownership of their care as much as possible and improve their own outcomes [22]. Therefore, considering the importance of this issue and due to the lack of evidence on an educational model based on the nursing process in Iran, the present study was conducted to determine the effect of education based on the nursing process on knowledge and self-care performance of elderly patients with surgical stoma.

## 2. Materials and Methods

### 2.1. Study Design, Setting, and Participants

This quasi-experimental study was conducted by using a pretest-post test two-group design. The population of this study was the elderly patients with an intestinal ostomy (ileostomy or colostomy) who were referred to Razi Hospital in Rasht, in 2020–2021. This hospital is the largest public and educational hospital in Guilan province in terms of size, which provides the most diverse general, specialized, and subspecialized services in the field of internal diseases and surgery to its patients.

Sampling was done by a simple random method. In this way, the samples were allocated from the list of intestinal stoma surgery candidates who met the inclusion criteria and were placed one in between the control and intervention groups. For example, the first patient from the list of surgical candidates was allocated in the intervention group and the second patient from the list was allocated in the control group. To determine whether the first patient will be in the control group or in the intervention group, a random lottery method by flipping a coin was used.

The inclusion criteria included the following: elderly patients who were 60 years or older with intestinal ostomy were willing to participate in the study and had not participated in similar programs before. They should be literate to speak and write Persian fluently, had no visual or auditory problems (they should not have problems communicating), did not have a psychological disorder (scored 8 or higher above the 10-item AMT instrument), were able to perform daily life activities (getting a score of 12 or higher assessed by using daily life tool), getting a score of 17 and lower (average and poor knowledge level) assessed by the Stoma Care Knowledge Level Questionnaire, earning a score of 18 and below (average performance level and poor) assessed by the checklist of checking the level of ostomy care performance and lack of work experience in the health system. The data collection took place from August 2020 to February 2021 and lasted for 7 months.

### 2.2. Research Instruments

Research instruments included a demographic characteristic questionnaire (including age, sex, and education), an ostomy self-care knowledge questionnaire, and an observational checklist of patients' ostomy self-care performance. The stoma care knowledge questionnaire developed by researchers is based on scientific text [25]. In this way, according to the objectives of the study, appropriate items were designed. Then, the phrasing, sequence, and arrangement of the questions and the proper format to receive the answers were considered. This questionnaire was related to how to take care of the ostomy (ileostomy and colostomy) and evaluates items such as the definition of ostomy, definition of ileostomy and colostomy, types of ostomy bags, duration of use of bags, appropriate time to change the bag, diet, lifestyle with ostomy, and stoma care tips. This questionnaire has 26 questions with “correct,” “incorrect,” and “I do not know” answers and is in 5 dimensions. For each correct answer, 1 point, and for incorrect and “I do not know” answers, zero points were considered. The sum of the total correct answers was 26. That if they got a score of 18 to 26, had good knowledge; 10 to 17, had average knowledge; and a score of 0 to 9, had poor knowledge. People with average to poor knowledge entered the training program. The questionnaire was answered one day before surgery as a pretest, and the first day, one week and one month after surgery as a posttest. The validity of the ostomy knowledge level questionnaire was confirmed using content validity (CVI and CVR) and its reliability was determined 0.85 by Kuder–Richardson 21 (KR-21). In this way, the questionnaire was sent to 10 faculty members of the gastroenterology, cancer surgery, and nursing departments of Guilan University of Medical Sciences, and their opinion regarding the importance of questionnaire items was obtained by selecting one of the options of relevance, clarity, simplicity, and necessity. After applying the professors' opinions, the final questionnaire was prepared. In the reporting phase, the questionnaire was sent to all professors and their approval was obtained.

The observational checklist of patients' ostomy self-care performance was taken from a book about basic nursing techniques. This procedure checklist book applied all the procedures from Craven and Hirnle's Fundamentals of Nursing [26]. This observational checklist has 13 items with a three-choice Likert rating of “excellent performance,” “satisfactory performance,” and “need to practice,” which evaluates the steps of changing the ostomy bag. For scoring, 2 points for “excellent performance,” 1 point for “satisfactory performance,” and zero for “need to practice” were considered. The total result was 26 because 13 items were examined. If the participants got a 18–26 score, their performance was excellent; 10 to 17 was used for average performance; and a score of 0 to 9 for poor performance. People with average to poor performance entered the training program.

To evaluate the performance of both groups in terms of how to change the bag and wash the ostomy, a pretest was performed on the day before surgery. An observation checklist of self-care performance was used for evaluation, which was completed by one of the researchers.

To evaluate the performance of both groups in terms of how to change the bag and wash the ostomy, a pretest was performed on the day before surgery. An observation checklist of self-care performance was used for evaluation, which was completed by one of the researchers. Then, changing the bag and washing the ostomy were practically taught in a session and the performance evaluated through posttest 1 on the first day, posttest 2 in one week after the training, and posttest 3 in one month after the training.

The validity of the observational performance checklist was confirmed using content validity, and its reliability was determined by an inter-rater reliability method (*r* = 0.9). Charlson Comorbidity Index was used to control concomitant diseases in the present study.

### 2.3. Intervention

The educational intervention in this study adopted five stages of the nursing process: assessment and recognition of educational needs, nursing diagnoses, planning, implementation of the educational program, and evaluation of the educational outcome [22, 27]. The first stage of the nursing process is assessing and recognizing the educational need, which was done using a questionnaire to assess the level of knowledge of ostomy care. After the necessary information was collected in the nursing assessment stage, nursing diagnoses were designed in the next stage. These nursing diagnoses were in the fields of patient knowledge related to nutrition, activity, and exercise, how to change the colostomy and ileostomy bag and the right time to change the bag, signs of the need to see a doctor or nurse, and important points of ostomy care. Then, in the third stage of the nursing process (planning), educational goals were formulated and educational content was prepared to achieve the goals.

The fourth stage of the nursing process was the implementation of educational intervention. Patients were trained individually and face-to-face. The training sessions were conducted in two sessions. The first training session was conducted one day before surgery with the aim of assessment of the patient's knowledge about ostomy care. Knowledge of ostomy care includes the definition of ostomy; the types of ostomy bags; proper nutrition; time to change the bag and the amount of activity, exercise, and symptoms required to see a doctor or nurse; and important ostomy care tips. An educational pamphlet was also provided for each patient.

In the evaluation process of the nursing process, the questionnaire was answered by a pretest and post-test by the subjects. For posttest, a questionnaire to assess the level of ostomy care knowledge was distributed and completed by the subjects one day after training, one week and one month after training. The second session of postoperative training was performed on the first bag replacement and ostomy lavage. In this session, the patient was taught how to take care of the ostomy and change the bag in a practical way. The duration of training was 15 minutes.

In the evaluation phase of the nursing process, the performance of the subjects in relation to how to change the bag and wash the ostomy as a pretest and post-test one day after training on how to care for the ostomy and how to change the bag, one week and one month after training with checklist.

In order to collect data, the researcher obtained informed consent after selecting the research samples and introducing herself and providing sufficient explanations about the purpose of the research. The participants were also reminded that the information obtained from them will remain completely confidential and they can withdraw from the study whenever they wish.

### 2.4. Data Analysis

Collected data were analyzed using the SPSS software version 21. To analyze the general data, descriptive statistics of mean, median, and standard deviation were used. The Kolmogorov–Smirnov (KS) test was used to investigate the normality distribution of quantitative variables. The Mann–Whitney *U* test was used to analyze the differences in the performance status and knowledge status between the two groups of the study, and Wilcoxon test was used to analyze the differences within the group of the study. The significance level (*p* value) in all tests was considered 0.05.

## 3. Results

The results showed that the majority of allocated participants was in the age group of 60–74 (86.5%) and were males (65.4%). All allocated participants (100%) were married. There was no statistically significant difference between the demographic characteristics of the two groups (*p* < 0.05).

The results related to the level of self-care knowledge showed that the mean score of knowledge increased one day and one week after the intervention in both intervention and control groups. At the end of the study, after thirty days, the self-care knowledge score of the intervention group had a higher median and mean than the control group (*p* < 0.001) (Table 1).

**Figure 1:**
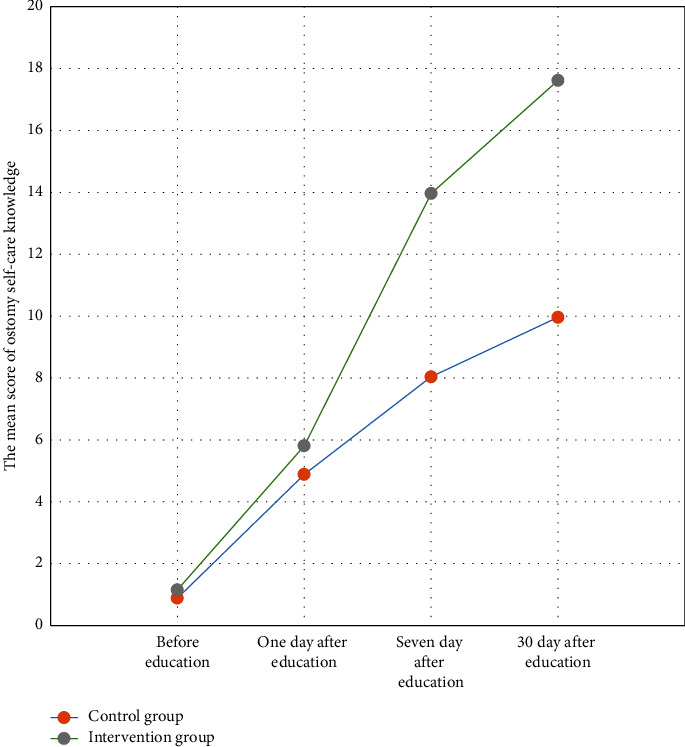
The mean score of ostomy self-care knowledge throughout the timeline of the study.

Figure 1 indicates that although both groups of the study improved their level of knowledge throughout the timeline of the study, the rate of promotion in the intervention group was more significant than the control group.

The results showed that the mean score of patients' performance in all time intervals (one day, one week, and one month after training) in the intervention group was higher than the control group (Table 2).

**Figure 2:**
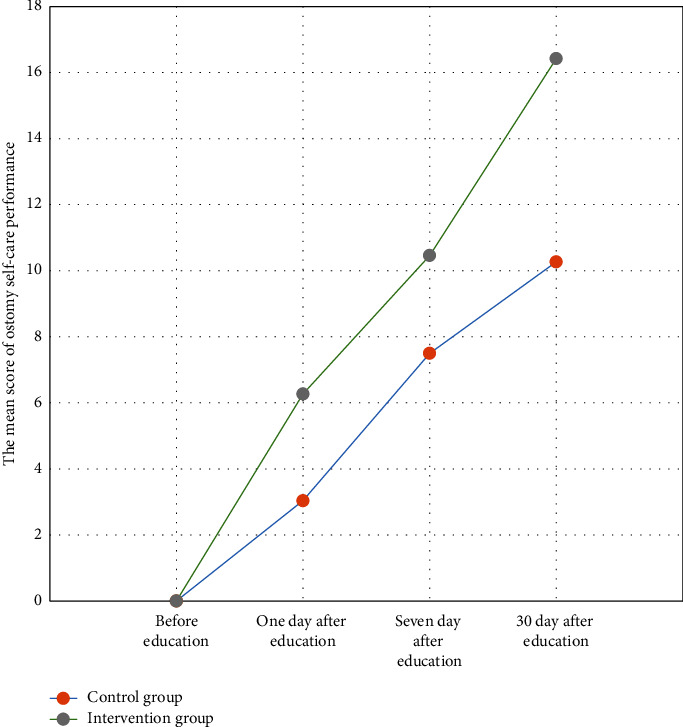
The mean score of ostomy self-care performance in different distinct times.

Figure 2 shows the mean score of ostomy self-care performance in both groups. It indicates that although both groups had improved performance levels during the study, the rate of promotion in the intervention group was significantly higher.

## 4. Discussion

This study was performed to determine the effect of education based on the nursing process on self-care knowledge and performance of elderly patients with intestinal stoma. Findings about self-care knowledge and performance in the intervention group and control group before and after the intervention showed that although both groups had improved the level of knowledge and performance, the percentage of promotion in the intervention group was significantly higher. In relation to the justification of this finding, it can be said that older adults are more at risk of developing chronic conditions. Thus, their search for health care will likely also increase [28]. This makes them look for the necessary information to take care of themselves, which can be a justification for improving the level of knowledge and performance in both the groups in the present study. However, the percentage of higher improvement in the level of knowledge and performance among the intervention group compared to the control group is related to the effect of the intervention. In other words, the results of the present study indicate that education based on the nursing process has been effective in improving self-care knowledge and performance. In justification of this finding, there was not found a study similar to the present study that investigated the intervention of education based on the nursing process on the level of knowledge and performance of patients with ostomy. For this reason, to discuss the findings, studies that have generally dealt with the effect of education on the self-care of these patients have been mentioned as evidence. For example, Shrief and Mokhtar examined the effect of structural education on the knowledge of patients with surgical stoma [24]. Consistent with the present study, the results of this study indicate the effect of education on self-care knowledge of patients with surgical stoma. Similar results were observed in the study of Ran et al. [29], Culha et al. [16], Hegazy et al. [30] and Oshvandi et al. [31]. In line with the results of the present study, the findings of the study by Pouresmail et al. [32], Hegazy et al. [30], and Sanabadi et al [33] showed the positive effect of education on increasing self-care skills and behaviors.

In general, adult education about various health care topics is essential to improve health care behavior and self-management as well as reduce risky behavior [34]. The ultimate goal of patient educational programs is to achieve long-lasting changes in behavior by providing patients with the knowledge to allow them to make autonomous decisions to take ownership of their care as much as possible and improve their own outcomes [22]. A great deal of effort must be invested to support the empowerment of older people to increase their benefits from educational interventions. To achieve empowerment, people must obtain knowledge that is related to individual needs and expectations. By providing empowering care, nurses help increase the residents' independence and their feelings of autonomy [34]. Gaining insight into older patients' needs, priorities, and experiences is also mentioned and of high importance in reviews on principles of learning in older people [35]. In older people, age-related conditions and the related decline in physical and cognitive functions require that they receive appropriate education to continue living as actively and independently as possible [34].

The nurse, as a part of the multiprofessional health team, has the duty of managing the care of elderly people with an intestinal ostomy, from any levels of assistance, from the diagnosis process to home monitoring [36]. Nurses are required to be adequately prepared with a sufficient theoretical knowledge base which guides their clinical practices [37] and provision of comprehensive nursing care to older adults [38]. The nursing process has become the basis of contemporary practice, as a core component of nursing education, as well as a point of reference in providing nursing care in many parts of the world. Arguably, it is central to all nursing actions, applicable in any setting and within any frame of [39]. The nursing process functions as a systematic guide to client-centered care [40]. Using the five-step nursing process for developing a patient teaching plan will help to deliver comprehensive and effective teaching. Simple teaching plans accompanied by multiple teaching strategies provide patients with valuable information [27]. The nursing process is regulated and implemented according to the needs of patients and due to the fact that implementation of care programs based on patients' needs will produce better results [41], it is expected that more attention will be paid to it in patient care plans.

## 5. Conclusion

Generally, the results of the study indicate that the use of education based on the nursing process has a positive effect on the self-care knowledge and performance of elderly patients with intestinal stoma and can be used as a model for nursing interventions in caring for patients. Therefore, it is suggested that education based on the nursing process has been considered in clinical and educational environments, and the relevant authorities should provide the necessary resources and facilities for nurses to use them.

The main limitation of the present study is that it was conducted on elderly patients of one medical center of Guilan University of Medical Sciences, which may not be representative of all older adults, and therefore, its results cannot be generalized to all older adults. It is suggested that future studies be conducted on participants from other regions and countries with cultural diversity and their results be compared with the findings of the present study.

## Figures and Tables

**Table 1:** The comparison of the mean score of ostomy self-care knowledge between the intervention and control groups after the completion of the programme.

Variables	Study groups						Sig
	Intervention group			Control group			
Increase in the score of ostomy self-care knowledge	Mean	SD	Median	Mean	SD	Median	
One day after intervention	4.65	3.11	4.00	4.00	1.74	4.00	0.356
7 days after intervention	12.81	3.14	12.00	7.15	3.38	7.00	0.000
30 days after intervention	16.46	2.60	17.00	9.08	2.92	8.00	0.000

SD: standard deviation; Sig: significance level.

**Table 2:** Comparison of the increase in the score of ostomy self-care performance in the two groups of the study.

Variables	Study groups						Sig
	Intervention group			Control group			
Increase in the score of ostomy self-care performance	Mean	SD	Median	Mean	SD	Median	
1 day after intervention	6.27	3.32	6.50	3.04	3.42	2.00	0.000
7 days after intervention	10.46	6.02	12.00	7.50	3.48	8.00	0.035
30 days after intervention	16.42	7.48	18.00	10.27	5.77	8.50	0.002

SD: standard deviation; Sig: significance level.

## Data Availability

The data set generated in this study is available upon reasonable request from the corresponding author.
